# Supplementary material for: Comparative Transcriptome Analyses of Leg Muscle during Early Growth between Geese (Anser cygnoides) Breeds Differing in Body Size Characteristics
Source: Genes (Basel). 2023 May 7;14(5):1048. doi: 10.3390/genes14051048 (PMC10217984; doi:10.3390/genes14051048)
Supplement: Supplementary file 1 [file genes-14-01048-s001.zip › Table S1ú║The primers for qPCR validation.pdf]

Table S1 The primers for qPCR validation

Accession NO.	Gene symbol	Primer sequence	Anneaning temperature	Product size
XM_013180350.1	EIF4E3	F: CAACGCCAAGGGAGGTAT<br>R: TCACGAACGCTGACGCTA<br>R: CACATTCCACCACCAACT	50℃	153 bp
XM_013172551.1	LOC106030656	F: GCCCAATGTAACTATCAG<br>R: TCTTTGCCATCCCACTTC	49℃	192 bp
XM_013186777.1	MAP3K4	F: ATTCCCCACCTTGCTGTGAG<br>R:GACTCGGCAAAGGACCAAGA	55℃	107 bp
XM_013201113.1	MYLK4	F: AAGGAGCTGGACATGGTT<br>R: TCTGTTGAATCTGGAGGT	44℃	157 bp
XM_013196238.1	NDUFA7	F: CCAGCCCTATTTGTGAGA<br>R: AAGTTCCATACAGCAGCATT	46.7℃	119 bp
XM_013172615.1	ODC1	F: ACGTTAGGTGCAGTCAGA<br>R: CACAGAAGGACTTGGGTA	59℃	106 bp
XM_013202142.1	SLC2A1	F: TTGGGTTTGTGGCGTTCT<br>R: GACAGCCCAGCAACAGCA	59℃	112 bp
XM_013183629.1	SLC25A5	F: GACCCAAAGAACACCCAC<br>R: GACGACGAACTGTATCAAAA	65℃	100 bp
XM_013191136.1	SSTR4	F: TGTTTGTGGTCTGCTGGATG<br>R: AGGATGAGGGAGGCGTTG	59℃	100 bp
XM_013195871.1	CXCL12	F: TTACCGATGTCCCTGTC<br>R: TTATGAGTGCGATGACG	44℃	207 bp
XM_013198276.1	ANKRD9	F: CGCTGGCAGGACCTCTTAG<br>R: GAAGTGCTCGGGAGAAATGG	49.7℃	117 bp
MG674174	GAPDH	F:TCTGTCGTGGACCTGACCTGC<br>R:GCCAGCACCCGCATCAAA	56℃	180bp
