# Supplementary material for: Comparative Transcriptome Analyses of Leg Muscle during Early Growth between Geese (Anser cygnoides) Breeds Differing in Body Size Characteristics
Source: Genes (Basel). 2023 May 7;14(5):1048. doi: 10.3390/genes14051048 (PMC10217984; doi:10.3390/genes14051048)
Supplement: Supplementary file 1 [file genes-14-01048-s001.zip › Table S2ú║Results of the estimates for the three nonlinear models.pdf]

Table S2. Results of the estimates for the three nonlinear models

Items	Goose	Model	A	B	K	R^{2*}	WGI[a]	BGI[b]
Body weight, g	Shitou goose	Logistic	5290.870	37.334	0.608	0.999	5.954	2645.435
		Bertanlanffy	6252.152	1.115	0.258	0.995	4.680	1852.489
		Gompertz	5813.700	5.935	0.346	0.998	5.147	2138.741
	Wuzong goose	Logistic	2692.975	23.443	0.638	0.998	4.944	1346.488
		Bertanlanffy	2984.940	0.983	0.303	0.995	3.569	884.427
		Gompertz	2866.950	4.746	0.382	0.997	4.077	1054.692
Body length, cm	Shitou goose	Logistic	41.720	4.421	0.344	0.995	4.321	20.860
		Bertalanffy	50.563	0.485	0.153	0.991	2.451	14.982
		Gompertz	46.813	1.886	0.201	0.992	3.157	17.222
	Wuzong goose	Logistic	30.580	3.182	0.376	0.987	3.078	15.290
		Bertalanffy	33.649	0.420	0.204	0.989	1.133	9.970
		Gompertz	32.483	1.568	0.247	0.989	1.821	11.950
Chest deep, cm	Shitou goose	Logistic	13.486	3.724	0.369	0.987	3.563	6.743
		Bertalanffy	15.305	0.445	0.184	0.978	1.570	4.535
		Gompertz	14.570	1.697	0.230	0.981	2.299	5.360
	Wuzong goose	Logistic	10.982	3.041	0.365	0.998	3.047	5.491
		Bertalanffy	12.089	0.409	0.198	0.995	1.033	3.582
		Gompertz	11.666	1.520	0.240	0.996	1.745	4.292
Chest width, cm	Shitou goose	Logistic	16.465	5.411	0.350	0.995	4.824	8.233
		Bertalanffy	20.946	0.523	0.143	0.986	3.150	6.206
		Gompertz	18.921	2.090	0.195	0.989	3.780	6.961
	Wuzong goose	Logistic	11.972	3.966	0.388	0.996	3.551	5.986
		Bertalanffy	13.421	0.457	0.197	0.990	1.602	3.977
		Gompertz	12.844	1.758	0.244	0.992	2.312	4.725
Pelvis width, cm	Shitou goose	Logistic	11.298	3.569	0.409	0.980	3.111	5.649
		Bertalanffy	12.439	0.428	0.213	0.965	1.174	3.686
		Gompertz	11.994	1.629	0.261	0.969	1.870	4.412
	Wuzong goose	Logistic	8.580	2.603	0.445	0.987	2.150	4.290
		Bertalanffy	8.998	0.372	0.270	0.980	0.406	2.666
		Gompertz	8.850	1.362	0.313	0.982	0.987	3.256
Keel length, cm	Shitou goose	Logistic	21.676	6.438	0.329	0.993	5.660	10.838
		Bertalanffy	30.941	0.566	0.116	0.987	4.564	9.168
		Gompertz	26.462	2.306	0.169	0.989	4.944	9.735
	Wuzong goose	Logistic	15.762	4.691	0.349	0.994	4.429	7.881
		Bertalanffy	18.558	0.501	0.165	0.995	2.469	5.499
		Gompertz	17.397	1.962	0.211	0.995	3.194	6.400
Tibia length, cm	Shitou goose	Logistic	10.385	2.387	0.451	0.992	1.929	5.193
		Bertalanffy	10.836	0.354	0.278	0.982	0.216	3.211
		Gompertz	10.677	1.286	0.321	0.985	0.784	3.928
	Wuzong	Logistic	8.499	1.891	0.504	0.997	1.264	4.250

	goose	Bertalanffy	8.700	0.314	0.339	0.991	-0.176	2.578
		Gompertz	8.633	1.110	0.380	0.993	0.275	3.176
Shank circumference, cm	Shitou goose	Logistic	5.835	1.982	0.420	0.994	1.629	2.918
		Bertalanffy	6.079	0.323	0.266	0.987	-0.118	1.801
		Gompertz	5.995	1.149	0.304	0.990	0.457	2.205
	Wuzong goose	Logistic	4.405	1.529	0.554	0.996	0.766	2.203
		Bertalanffy	4.474	0.278	0.393	0.996	-0.462	1.326
		Gompertz	4.452	0.965	0.432	0.996	-0.082	1.638
Semi-submersible length, cm	Shitou goose	Logistic	80.536	4.030	0.348	0.998	4.005	40.268
		Bertalanffy	94.427	0.466	0.165	0.994	2.031	27.978
		Gompertz	88.696	1.791	0.211	0.996	2.762	32.629
	Wuzong goose	Logistic	61.177	3.128	0.391	0.997	2.917	30.589
		Bertalanffy	66.381	0.413	0.217	0.995	0.988	19.668
		Gompertz	64.419	1.543	0.261	0.996	1.662	23.698
Neck length, cm	Shitou goose	Logistic	28.192	3.455	0.369	0.996	3.360	14.096
		Bertalanffy	31.365	0.436	0.195	0.995	1.377	9.293
		Gompertz	30.112	1.645	0.239	0.996	2.083	11.078
	Wuzong goose	Logistic	21.191	2.891	0.429	0.996	2.475	10.596
		Bertalanffy	22.512	0.397	0.251	0.995	0.696	6.670
		Gompertz	22.029	1.470	0.295	0.996	1.306	8.104

[*]R^2 represents the goodness of fit.

[a]WGI indicates the weeks of age at the inflexion point of growth.

[b]BGI indicates the value at the inflexion point of growth.
